# Neonatal Late-Onset Sepsis Due to Acinetobacter baumannii: A Case Report

**DOI:** 10.7759/cureus.74108

**Published:** 2024-11-20

**Authors:** Marta Oliveira Martins, Rita Marchante Pita, Mayara Nogueira, Ana Ferraz, João R Martins

**Affiliations:** 1 Pediatrics Department, Unidade Local de Saúde de Coimbra, Coimbra, PRT; 2 Neonatology Department, Maternidade Daniel de Matos, Unidade Local de Saúde de Coimbra, Coimbra, PRT

**Keywords:** late onset neonatal sepsis, mdr acinetobacter baumannii, multi-drug resistance, neonatal intensive care unit, acinetobacter baumannii

## Abstract

Late-onset sepsis (LOS) is commonly associated with pathogens acquired in hospital or community settings and carries a significant risk of morbidity and mortality in neonates.

We present a case of a late preterm neonate, born at 36 weeks and 2 days with low birth weight (1700 g), who was admitted to the neonatal intensive care unit (NICU) and developed LOS on the fourth day of life. LOS was diagnosed in the context of fever and lethargy, mild thrombocytopenia, leukopenia, and lymphopenia, and was caused by multidrug-resistant (MDR) *Acinetobacter baumannii*, confirmed through blood culture. Initial empirical treatment with vancomycin and gentamicin was adjusted to gentamicin and meropenem based on the antibiogram, resulting in a swift and favorable clinical and laboratory response.

This case, a rare occurrence in our NICU, provides an opportunity to discuss the etiology, risk factors, and clinical presentation of LOS related to *Acinetobacter* *baumannii,* as well as to highlight the growing concern of MDR microorganisms in the NICU setting.

## Introduction

Late-onset sepsis (LOS) typically occurs after three to seven days of life [1], with an incidence of up to 21% [2]. In neonatal intensive care units (NICU), coagulase-negative *Staphylococci* are the most frequently identified pathogens in neonates with LOS [1], followed by *Escherichia coli*, group B *Streptococcus*, other Gram-negative aerobes, and *Listeria monocytogenes* [1,2]. Among these, *Acinetobacter (A.) baumannii*, though less common, has emerged as a notable pathogen in neonatal intensive care unit (NICU) settings, particularly in cases of neonatal septicemia and severe illness. The reasons for this rise are not fully understood, but host, pathogen, and environmental factors all likely play a role [3].

*A. baumannii* is increasingly recognized for its role in bloodstream and respiratory infections and has been associated with hospital outbreaks globally [4-6]. The prevalence of *Acinetobacter *spp. in neonatal sepsis varies widely worldwide, ranging from 1-6% of culture-positive cases, with higher rates observed in Central Africa (11%) and India, where studies report up to 22% in certain areas [3]. This pathogen can lead to severe disease, particularly in vulnerable populations such as preterm infants, low birth weight (LBW) infants, and those requiring assisted ventilation, vascular devices, or prior antibiotic exposure [6]. Additional risk factors include preterm rupture of membranes, chorioamnionitis, meconium aspiration, and maternal fever or infections during labor [1,2].

Furthermore, *A. baumannii* poses a unique threat due to its high resistance to multiple antibiotics. Resistance rates of up to 70% to gentamicin, amikacin, and carbapenems, and 84% to third-generation cephalosporins have been reported [3].

Prompt administration of antibiotics and supportive care is crucial, as *A. baumannii *LOS carries high rates of morbidity and mortality [2].

## Case presentation

We report the case of a female preterm newborn, born to a 31-year-old mother with no notable health conditions. The pregnancy was closely monitored and complicated by preeclampsia and fetal growth restriction, which was detected at 33 weeks of gestation. At 36 weeks and 2 days, a cesarean section was performed due to maternal clinical and laboratory deterioration related to preeclampsia. The procedure was uneventful, and the newborn adapted well to extrauterine life, with Apgar scores of 9/10/10 and a birth weight of 1700 g (low birth weight (LBW)). Following delivery, she was admitted to the NICU for close monitoring.

On the fourth day of life, the newborn developed a fever and lethargy, raising suspicion of LOS. Laboratory investigation, as presented in Table 1, revealed mild thrombocytopenia (114000/µL), mild leukopenia (4200/µL), and mild lymphopenia (1250/µL) while C-reactive protein and procalcitonin levels remained within normal limits. A blood culture sample was also obtained. Although the neonate was hemodynamically stable and maintained spontaneous breathing without additional symptoms, close monitoring was maintained, and empirical antibiotic therapy with vancomycin and gentamicin was promptly initiated due to clinical suspicion of LOS.

**Table 1:** Laboratory investigation including a complete blood count, inflammatory parameters, and blood culture

Parameters	Patient values	Reference range
Leucocytes	4200/µL	5000 – 21000/µL
Neutrophils	2630/µL	1500 – 15000/µL
Lymphocytes	1250/µL	2000 – 17000/µL
Monocytes	260/µL	200 – 2500/µL
Eosinophils	60/µL	40 – 1000/µL
Basophiles	20/µL	0 – 250/µL
Erythrocytes	4.25 x 10^12/L	3.9 – 6.3 x 10^12/L
Haemoglobin	15.2 g/dL	13.5 – 21.5 g/dL
Platelets	114000/µL	150000 – 490000/µL
C-reactive protein	0.22 mg/dL	< 0.50 mg/dL
Procalcitonin	0.37 ng/mL	0 – 0.5 ng/mL
Blood culture	Positive: Acinetobacter baumannii detected	

Direct observation of the blood sample indicated the presence of Gram-negative bacilli and subsequent culture-confirmed *Acinetobacter baumannii.* Additionally, *A. baumannii* was identified through rapid multiplex polymerase chain reaction-based blood culture identification.

Ceftazidime was added to the treatment regimen. Based on the antibiogram results, presented in Table 2, therapy was adjusted to meropenem and gentamicin. This treatment was maintained for eight days.

**Table 2:** Antibiogram of the Acinetobacter baumannii detected in the blood culture

Antibiotics	Level of sensitivity
Cefotaxime	Resistant
Meropenem	Susceptible
Levofloxacin	Susceptible
Imipenem	Susceptible
Gentamicin	Intermediate (susceptible with increased exposure)
Ciprofloxacin	Susceptible
Cefuroxime	Resistant
Cefazolin	Resistant

Contact and droplet isolation precautions were implemented. Serial analysis, presented in Table 3, showed a peak C-reactive protein level of 3.38 mg/dL and a procalcitonin peak of 5 ng/mL on the fifth day of life, both of which declined thereafter. The blood count normalized after 48 hours of antibiotic therapy. A follow-up blood culture, performed after eight days of treatment, was negative.

**Table 3:** Serial laboratory investigation including a complete blood count, inflammatory parameters, and blood culture

Parameters	Patient values				Reference range
	4^th^ day of life	5^th^ day of life, 24 hours of antibiotics	6^th^ day of life, 48 hours of antibiotics	12^th^ day of life, 8 days of antibiotics	
Leucocytes	4200/µL	12600/µL	9000/µL		5000 – 21000/µL
Neutrophils	2630/µL	8100/µL	3500/µL		1500 – 15000/µL
Lymphocytes	1250/µL	1400/µL	4000/µL		2000 – 17000/µL
Monocytes	260/µL	300/µL	130/µL		200 – 2500/µL
Eosinophils	60/µL	40/µL	40/µL		40 – 1000/µL
Basophiles	20/µL	0/µL	0/µL		0 – 250/µL
Erythrocytes	4.25 x 10^12/L	3.92 x 10^12/L	3.90 x 10^12/L		3.9 – 6.3 x 10^12/L
Haemoglobin	15.2 g/dL	13.9 g/dL	13.7 g/dL		13.5 – 21.5 g/dL
Platelets	114000/µL	54000/µL	160000/µL		150000 – 490000/µL
C-reactive protein	0.22 mg/dL	3.38 mg/dL	1.22 mg/dL		< 0.50 mg/dL
Procalcitonin	0.37 ng/mL	5 ng/mL	1.89 ng/mL		0 – 0.5 ng/mL
Blood culture	Positive: Acinetobacter baumannii detected			Negative	

The patient showed rapid clinical improvement, becoming afebrile and recovering within 24 hours of starting empirical antibiotic therapy. She maintained spontaneous breathing without requiring supplemental oxygen therapy or ventilatory support and remained hemodynamically stable.

The neonate was discharged on the nineteenth day of life after achieving oral feeding competence and remained clinically stable throughout her stay. A follow-up evaluation at one month of age revealed normal growth and development, with continued care provided through the National Program under her family doctor.

## Discussion

*Acinetobacter baumannii *is rarely reported as a cause of neonatal sepsis in developed countries, although it is relatively more frequent in certain regions of the world [7-9]. In these areas, it has emerged as a significant cause of healthcare-associated infections and hospital outbreaks, particularly among critically ill patients in NICUs [4,5]. In this case, several factors support the diagnosis of LOS due to *A. baumannii*: the presence of clinical signs such as fever and lethargy; identification of *A. baumannii* in blood culture; LBW and preterm birth, known risk factors for LOS; and improvement after administration of appropriate antibiotics, suggesting an active infection rather than contamination.

Although rare in some settings, *A. baumannii* is associated with substantial morbidity and mortality, largely due to its propensity for antimicrobial resistance [9]. *Acinetobacter spp.* strains often exhibit resistance to the recommended first-line empirical treatment for neonatal sepsis such as ampicillin and gentamicin. The need for escalation to broader-spectrum antibiotics has contributed to the emergence of more resistant strains, including carbapenem-resistant strains, leading to infections that are increasingly challenging to manage [3]. In the latest report from the World Health Organization (WHO), *A. baumannii *is listed as a critical priority for research and development of public health measures, particularly due to its ability to transfer resistance genes, the severity of the infections and diseases it causes, and its significant global burden [10].

Neonates with *Acinetobacter spp.* sepsis often experience higher rates of complications, including a greater need for mechanical ventilation, vasoactive drugs, and poorer survival rates [8], particularly in cases involving MDR strains [6]. The global rise in antibiotic-resistant *Acinetobacter* strains has led to increasing concerns regarding potential epidemics and hospital outbreaks [6]. Notably, *A. baumannii* is now recognized as one of the leading pathogens contributing to deaths associated with antimicrobial resistance [11].

In our NICU, this was the first case of *A. baumannii* sepsis. Fortunately, the clinical course was favorable, with no need for ventilatory or circulatory support. Early detection and timely administration of appropriate antibiotic therapy likely played a crucial role in preventing the deterioration of the newborn. In our case, prematurity and LBW were the only well-established risk factors identified.

This case is consistent with reports of a global increase in cases caused by this microorganism. The emergence of *A. baumannii *as a significant cause of neonatal sepsis is likely multifactorial. In NICU settings, where broad-spectrum antibiotics are commonly used to manage infections, resistant strains of *A. baumannii *can emerge and spread. However, other contributing factors should also be considered, such as inadequate adherence to infection control practices and the effects of globalization, including travel and the movement of healthcare professionals and patients.

The emergence of MDR bacteria in neonatal units is a serious concern that requires the attention of healthcare professionals. Prompt recognition and treatment of these infections are essential, and treatment regimens should be adjusted based on antibiogram results.

Simultaneously, it is imperative to reevaluate infection control protocols and implement preventive measures to reduce the occurrence of such infections. This includes stringent adherence to hand hygiene and equipment sterilization protocols, frequent disinfection of high-touch surfaces, minimizing the use of invasive devices whenever possible, judicious antibiotic use to reduce selective pressure, and the establishment of routine environmental sampling. Additionally, staff training in infection prevention and early identification of possible MDR organisms in the NICU are essential. Implementing active surveillance cultures and reporting mechanisms for MDR organisms in NICU can further enable early detection, allowing for timely intervention. Enhanced antibiotic stewardship programs, along with strict isolation practices for MDR cases, are also critical interventions to limit the spread and impact of MDR organisms.

## Conclusions

*Acinetobacter baumannii* is a rare yet significant cause of neonatal sepsis in developed countries, particularly in NICU settings where vulnerable preterm and low birth weight infants are at heightened risk. In comparison to studies from other regions where *A. baumannii* incidence is higher, this is the first reported case in our NICU. In terms of antimicrobial resistance, *A. baumannii *isolates in the NICU setting are often MDR, consistent with global trends of increased resistance to first-line antibiotics, and frequently, to carbapenems as well. To confirm *A. baumannii* as the pathogen rather than a contaminant, we emphasize the neonate’s clinical response to targeted therapy, the low likelihood of environmental contamination in blood culture, and the known virulence of MDR *A. baumannii*.

The limitations of this case report include the lack of maternal or environmental cultures, which could provide additional insights into the transmission pathway. This case underscores the importance of recognizing *A. baumannii *as a potential pathogen in cases of LOS, given its association with severe disease and multidrug resistance. Early detection and prompt, targeted antibiotic therapy are essential in managing these infections and preventing complications. With the growing prevalence of multidrug-resistant organisms in neonatal care, proactive measures, such as stringent infection control protocols, routine microbial surveillance, and judicious use of antibiotics, are essential. Our findings highlight the need for ongoing research into new antimicrobial treatments and enhanced infection prevention strategies to mitigate the impact of MDR pathogens like *A. baumannii *in neonatal units. We are likely to observe an increasing number of cases in the coming years, emphasizing the urgent need for the study and implementation of preventive strategies, as well as the development of novel antibiotic therapies.

## References

[1] Shane AL, Sánchez PJ, Stoll BJ (2017). Neonatal sepsis. Lancet.

[2] Ershad M, Mostafa A, Dela Cruz M, Vearrier D (2019). Neonatal sepsis. Curr Emerg Hosp Med Rep.

[3] Pillay K, Ray-Chaudhuri A, O’Brien S, Heath P, Sharland M (2024). Acinetobacter spp. in neonatal sepsis: an urgent global threat. Front Antibiot.

[4] Shete VB, Ghadage DP, Muley VA, Bhore AV (2009). Acinetobacter septicemia in neonates admitted to intensive care units. J Lab Physicians.

[5] Zarrilli R, Bagattini M, Esposito EP, Triassi M (2018). Acinetobacter infections in neonates. Curr Infect Dis Rep.

[6] Wei HM, Hsu YL, Lin HC (2015). Multidrug-resistant Acinetobacter baumannii infection among neonates in a neonatal intensive care unit at a medical center in central Taiwan. J Microbiol Immunol Infect.

[7] Mahich S, Angurana SK, Suthar R, Sundaram V, Munda VS, Gautam V (2021). Acinetobacter sepsis among out-born neonates admitted to neonatal unit in pediatric emergency of a tertiary care hospital in North India. Indian J Pediatr.

[8] Lee HY, Hsu SY, Hsu JF, Chen CL, Wang YH, Chiu CH (2018). Risk factors and molecular epidemiology of Acinetobacter baumannii bacteremia in neonates. J Microbiol Immunol Infect.

[9] Thomas R, Wadula J, Seetharam S, Velaphi S (2018). Prevalence, antimicrobial susceptibility profiles and case fatality rates of Acinetobacter baumannii sepsis in a neonatal unit. J Infect Dev Ctries.

[10] World Health Organization (2024). World Health Organization: WHO Bacterial Priority Pathogens List, 2024: bacterial pathogens of public health importance to guide research, development and strategies to prevent and control antimicrobial resistance. WHO Bacterial Priority Pathogens List, 2024: Bacterial Pathogens of Public Health Importance to Guide Research, Development and Strategies to Prevent and Control Antimicrobial Resistance.

[11] Antimicrobial Resistance Collaborators (2022). Global burden of bacterial antimicrobial resistance in 2019: a systematic analysis. Lancet.

